# Unicentric Castleman's disease approached as a pancreatic neoplasm: case report and review of literature

**DOI:** 10.1186/1757-1626-2-9090

**Published:** 2009-11-25

**Authors:** Adolfo Petrina, Emilio Eugeni, Marco Badolato, Carlo Boselli, Piero Covarelli, Fabio Rondelli, Giuseppe Noya

**Affiliations:** 1 Department of Surgical Sciences, Oncological Surgery Unit, University of Perugia, Italy

## Abstract

Castleman's disease is a rare lymphoproliferative disorder. Most cases occur in the mediastinum and the pancreatic localization is uncommon; currently there are only nine reported cases in the literature about peripancreatic localization. We report a case of a 62 years old man with a Castleman's disease mimicking a pancreatic neoplasm.

## Introduction

Castleman's disease (CD) or angiofollicular lymph node hyperplasia is a rare lymphoproliferative disorder of unknown aetiology first described in 1954 by B. Castleman and V.W. Towne as an asymptomatic benign hyperplastic lymph node resembling a thymoma[1,2]. Most of the lesions are located in the thorax (70%), but extrathoracic involvement (neck, axilla, mesentery and retroperitoneum) have also been reported [3-6].

When CD is localized in retroperitoneum it has no distinctive clinical or radiological features and it can be confused with other retroperitoneal tumours or, as in our description, with vascular anomaly. We report the case of a CD Tumour, mimicking a pancreatic mass and initially diagnosed as an aneurysm of the left gastric artery [7-10].

## Case report

**Figure 1:**
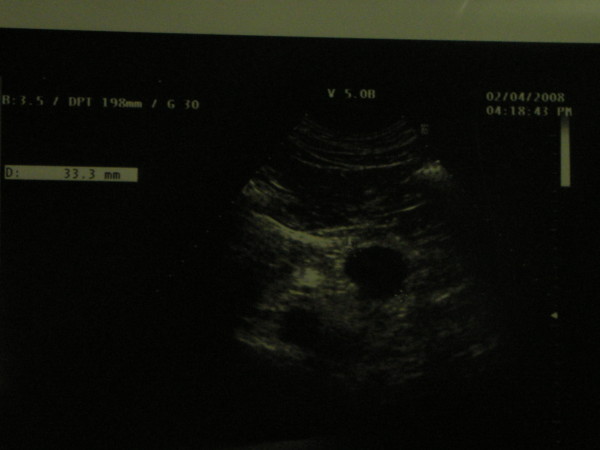
**Ultrasonography scan with occasional report of cystic mass of 38 × 28 mm localized in the pancreatic body**.

A 62 year old man with nephrolithiasis history underwent an ultrasonography scan (US) with occasional report of cystic mass of 38 × 28 mm localized in the pancreatic body, close to the tail (April 2nd, 2008, as shown in figure 1).

**Figure 2:**
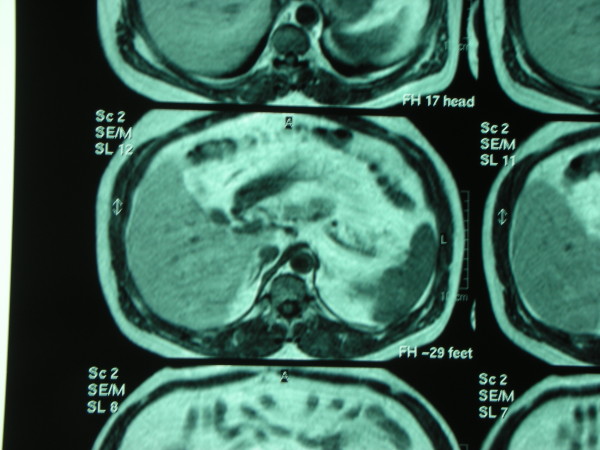
**MRI describing a mass of 38 mm localized in front of the pancreas body**.

The radiologist recommended a Magnetic Resonance Imaging (MRI) and a fine needle agobiopsy to characterize the lesion interpreted as a pancreatic cystoadenoma or a dysontogenetic cyst. The MRI (performed on April 22^nd ^- figure 2) described a mass of 38 mm localized in front of the pancreas body and behind the lesser gastric curvature, maybe a supernumerary spleen, this hypothesis to be confirmed with a Computed tomography (CT) scan.

The CT scan was performed on May the 7th: it revealed a fluid, roundish mass less than 40 mm of maximum diameter between lesser curvature and pancreatic body with contrast enhancement as arterial blood, perhaps aneurysm of left gastric artery.

**Figure 3:**
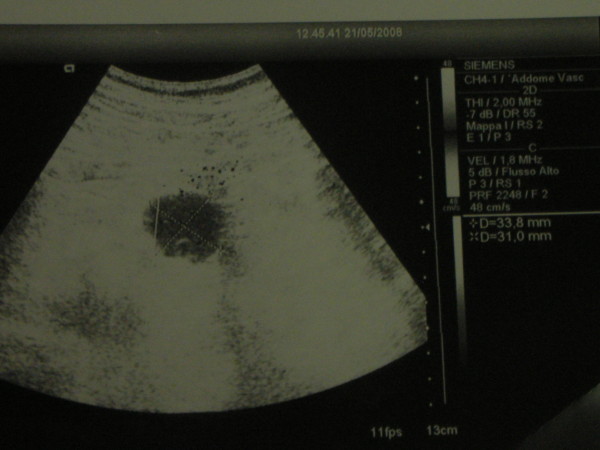
**Doppler sonography showing a thrombosis of a splenic artery aneurysm**.

A Doppler Sonography (May 21^st^, see figure 3) showed a thrombosis of a splenic artery aneurysm.

**Figure 4:**
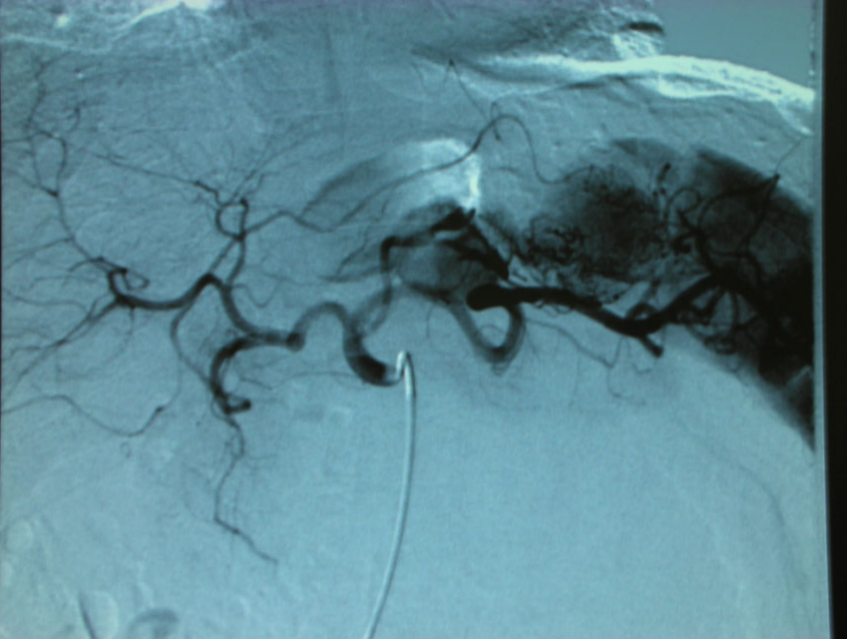
**Transfemoral arteriography demonstrating an aneurysm of the left gastric artery of 40 mm**.

The patient underwent on June 18th to a transfemoral arteriography that demonstrated an aneurysm of the left gastric artery of 40 mm not confirmed in a selective arteriography performed on June 24^th^; showed in figure 4.

Another doppler sonography on the same day gave evidence of a peripancreatic iso-anechogenic mass.

A MRI (July 7th) displayed a sovramesocolic mass between lesser curvature and gastric body: gastrointestinal stromal tumor (GIST) or hemangiopericytoma.

On September 12th a contrast-enhanced sonography testified an ipo-anechogenic mass of 36 mm in the pancreatic body as a not active neuroendocrinal neoplasm; on the same date an endo-ultrasonography deposed for an extragastric mass, with no certain cleavage with pancreas (the brown words do not make sense to me) of 36 mm of maximum diameter and satellite lymphadenopathy.

The patient was admitted to our unit on October 13th after all the exams and procedures described above and with no certain diagnosis. His past medical history showed type II diabetes mellitus, hypertension, hyperuricemia and hypertriglyceridaemia. Laboratory tests were normal except for mild hyperglicemia (117 g/dL) and a slight γ-GT increase (104 IU/L). There was no serological indication of active HIV, B and C hepatitis, past Epstein Barr virus and Cytomegalovirus infections and tumor markers as CEA and CA19.9 were normal. A colonoscopy was performed that showed a diverticulosis. The patient underwent surgical operation for suspected pancreatic neoplasm. At laparotomy the pancreas appeared normal as well as the liver and other organs. (It was found) A voluminous well vascularized 4 cm mass was found along the lesser gastric curvature, not contacting the gastric body or the pancreas. The laparotomy was restricted to dissect the mass. The postoperative course was uneventful and the patient was discharged on 5th day. Frozen section of the mass showed an atypical lymphoproliferative process with no clonal rearrangement of IgH gene at molecular studies; the final pathological report described an angiofollicular lymphoid hyperplasia compatible with mixed variety, hyaline-vascular plasma cell type of CD made of preserved lymphoid tissue with follicles at various degree of maturation and diffuse hyaline involution and intervening sheets of plasma cells and capillary.

He is alive and free of recurrence at 4 months follow-up.

## Discussion

Castleman's Disease usually presents in young adults with a median age of approximately 35 years, equally distributed between males and females; The cause of this disorder is not known and it may be caused by a faulty immunoregulation which results in the excessive proliferation of B lymphocytes and plasmacells in lymphoid organs; human Herpes virus-8 and Epstein Barr virus may play a role especially in immunocompromised hosts. According to Keller, McCarty, Gaba et al. 6 there are two types of CD, either a localized type (with a typical benign course), or a plurifocal, also called "multicentric", type which has usually malignant course [4,6,7]. In these two clinical entities have been described two distinct histological types and a mixed variant: the hyaline-vascular type, most frequent, and the plasmocytic type (10% of total cases). The former is clinically almost asymptomatic while the plasmocytic type is sometimes associated with systemic manifestation such as anaemia, weight loss, night sweats, fever and polyclonal hypergammaglobulinemia; it is also frequently associated with Acquired Immunodeficiency Syndrome, Kaposi sarcoma and POEMS syndrome (polineuropathy, organomegaly, endocrinopathy, M protein and skin changes) [7,8]. CD has no specific radiological features and it is almost indistinguishable from other disease and the features of neoplastic and non-neoplastic lesions and those with a malignant potential is poor. In a retrospective study by Bowne surgical excision has been associated with the best chance of cure for localised disease [10]; for multicentric disease the treatment has not been established due to its variable clinical course (chronic evolution with remission and exacerbation or rapidly progressive and fatal course) [7,9]. Corticosteroids, antimitotics, radiotheraphy and excision are, at the present, the therapeutic approaches. Surgery is both diagnostic and therapeutic and laparoscopic approach may be the gold standard for diagnosis and treatment [10]. Radiation therapy has been used with varied success in patients who are poor surgical candidates or in those with unresectable lesions.

A better understanding of the pathogenesis, natural history, and ultimately diagnosis of this disorder may lead to improvement over the current modalities available for treatment.

## Consent

The patient signed an informed written consent for publication of the manuscript and figures in it contained

## Competing interests

The authors declare that they have no competing interests.

## Authors' contributions

AP was a major contributor in writing the manuscript; EE analyzed and interpreted the patient data and clinical history; all authors read and approved the final manuscript
